# Potential Use of Natural Killer Cell Transfer Therapy in the Perioperative Period to Improve Oncologic Outcomes

**DOI:** 10.1155/2015/732438

**Published:** 2015-10-21

**Authors:** Juan P. Cata, Claudius Conrad, Katy Rezvani

**Affiliations:** ^1^Department of Anesthesiology and Perioperative Medicine, The University of Texas MD Anderson Cancer Center, Houston, TX 77030, USA; ^2^Anesthesiology and Surgical Oncology Research Group, Houston, TX, USA; ^3^Department of Surgical Oncology, The University of Texas MD Anderson Cancer Center, Houston, TX 77030, USA; ^4^Department of Stem Cell Transplantation, The University of Texas MD Anderson Cancer Center, Houston, TX 77030, USA

## Abstract

Immune suppression after oncologic surgery is a common phenomenon. Several studies have demonstrated that it is associated with poor survival owing to cancer progression. Immunotherapy, especially NK cell transfer therapy, is an attractive alternative because current methodologies to isolate, generate, and expand NK cells have shown good safety profiles in current active investigations. We believe that the use of NK cell transfer therapy in the context of postoperative minimal residual disease deserves significant investigation.

## 1. Introduction

The term immune surveillance refers to the immune system actively acting against the growth of developing tumors [1]. This process consists of 3 stages: elimination, equilibrium, and escape [2]. Natural killer (NK) cells are large, granular lymphocytes that participate in the process of elimination because they are able to efficiently destroy cancer cells [3]. NK cells can kill target cells that express low levels of major histocompatibility complex-I molecules, which otherwise would escape immune surveillance. The importance of NK cells in immune surveillance has been highlighted by experimental and clinical data showing that stimulation of NK cells protects against cancer metastasis and that a decrease in NK cell function enhances cancer metastasis [4–8]. Moreover, researchers have suggested that parameters of NK cell function could serve as prognostic biomarkers [9, 10].

On the basis of these findings, several investigators have proposed the use of NK cells in immunotherapy platforms. Immunotherapy can be described as the use of vaccines, immune adjuvants, cytokines, antibody-based therapy, or effector cells produced* in vitro* to improve immune surveillance and therefore achieve tumor control and cure [11]. Cell transfer therapies, especially NK cell-based transfer therapy (NKTT), have been investigated by medical oncologists for more than a decade for the treatment of hematologic and nonhematologic malignancies [12, 13]. It is worth mentioning that cell-based therapy for solid tumors was first proposed almost 30 years ago when lymphokine-activated killer cells were successfully administered to patients with melanoma [14].

Perioperative immune suppression has been reported after lung, ovarian, and brain cancer surgery [15–17]. Immune suppression is considered to be one of the main factors responsible for locoregional and distant metastasis after oncologic surgery [18]. Opioids, volatile anesthetics, surgical stress, and an imbalance between Th1 and Th2 cytokines have been implicated as the potential causes of postoperative immune suppression [19]. A particular characteristic of the perioperative immune suppression associated with oncologic surgery is a reduction in cell-mediated cytotoxicity, specifically diminished function or count of NK cells. The importance of adequate quantitative and qualitative NK cell biology in the perioperative period has been demonstrated in experimental models of cancer recurrence [20–22]. Specifically, the number of pulmonary metastases increased in rodents treated with anesthetics and analgesics, as well as surgery, which decrease the function of NK cells [20, 21].

Increasing evidence indicates that postoperative immune suppression plays a role in tumor progression, suggesting that immunotherapeutic strategies such as NKTT could be used in the future. Here, we review the current knowledge of NK cell biology in the perioperative period and the potential use of NKTT before, during, or immediately after surgery.

## 2. NK Cell Biology in the Perioperative Period

Human NK cells represent 5–15% of the total peripheral blood mononuclear cells, and they primarily originate from hematopoietic stem cells in the bone marrow. Trafficking and migration of NK cells into nonlymphoid organs is determined by the interaction of various soluble mediators with their receptors, including catecholamines, selectins (L-selectin), integrins, and chemokines (CXCR1, CXCR2, CXCR3 CXCR4, and CXCR6), as well as by signals induced by cytokines and sphingosine-1-phosphate [23].

**Figure 1:**
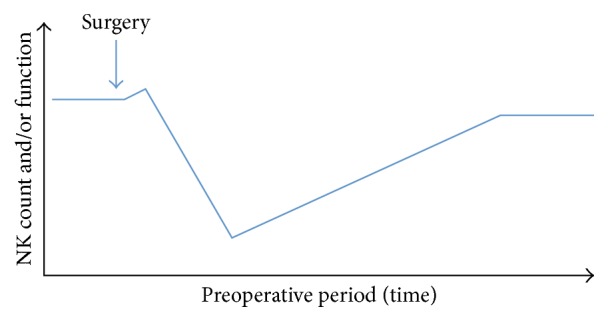
Commonly observed biphasic response in natural killer (NK) cell count and activity during and after surgery.

Although intense stress, such as that induced by short periods of exercise and acute pain, increases mobilization of NK cells in the blood, NK cell counts in the perioperative period appear to have a biphasic response that is similar to what is observed after prolonged exercise [24, 25]. Intraoperatively and minutes after surgery, the number of circulating NK cells surges, and this is mediated by the effect of epinephrine on *β*2-adrenergic receptors and a decrease in adhesion molecules [26, 27]. However, we and others have demonstrated that this initial rise in the number of NK cells appears to be followed by a sustained drop that might last several days or up to a month, as has been observed after surgery for stage II or III colorectal cancer (Figure 1) [15, 28–32].

**Figure 2:**
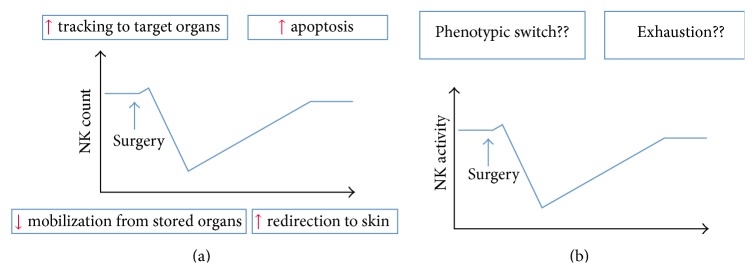
Postulated mechanisms behind the observed quantitative (a) and qualitative (b) changes in natural killer (NK) cells during and after surgery.

The reasons for the postoperative reduction in circulating NK cells are currently unknown, but it could be the result of (a) increased trafficking into target organs as a result of high circulating concentrations of cortisol; (b) increased rates of apoptosis also mediated by catecholamines; (c) reduced mobilization from the store organs into the blood as a result of prolonged *β*-adrenergic receptor stimulation, which has been shown in patients with chronic heart conditions; or (d) redirection of the cells to the skin, as proposed by Dhabhar et al. (Figure 2) [33–35]. Redirection of cells to the skin might in fact be the predominant phenomenon in the perioperative period because adrenergic stimulation reduces adhesion molecules in NK cells as well as the trafficking of NK cells from blood into organ tissues such as the lungs [26, 36].

Lastly, it is possible that the changes in mobilization or trafficking of NK cells in the perioperative period are the result of clinical interventions that can modulate the sympathetic system, such as administration of beta-blockers, nonsteroidal anti-inflammatory drugs, regional anesthesia, or a combination of these agents. For instance, it has been shown that epidural anesthesia and blockade of the stellate ganglion are associated with a qualitative and quantitative decrease in NK cells [37, 38]. Complicating the matter further, NK cell counts are also increased by other common intraoperative factors, such as hypovolemia and hypoxia [39, 40].

Phenotypically, NK cells can be divided into CD56^bright^ and CD56^dim^ cells. CD56^bright^ cells (approximately 10% of NK cells) are capable of enhanced proliferation and cytokine production, and CD56^dim^ cells display a more potent cytotoxic function [41]. NK cells exert their cytolytic activity (a) through direct effects on cancer cells and the release of cytotoxic granules containing granzymes (A, B, H, K, and M) and perforins, or (b) via the secretion of various cytokines such as interferon-*γ* and tumor necrosis factor-*α* [42, 43].

In addition to killing target cells through direct effects, as in the case of K562 cells, NK cells can trigger cancer cell death through the recognition of antibodies (NK-mediated antibody-dependent cellular cytotoxicity), as in the case of Raji cells. In both circumstances, the magnitude of the cytolytic activity of NK cells is the result of a complex intracellular signaling balance provided by activating or inhibiting receptors. The NKG2D receptors, DNAM-1 receptors, and natural cytotoxic receptors trigger the activation of NK cells, and the importance of these receptors in immune surveillance has been reported in experiments in which overexpression of the receptor on the surface of NK cells causes tumor rejection and, in patients who have achieved complete clinical remission, a return to normal levels of natural cytotoxic receptors after treatment [44, 45].

NK cells can also be activated by recognition of specific antigens via CD16, a low-affinity receptor for the Fc portion of immunoglobulin. Activation of NK cells via CD16 is characterized by an increase in the release of cytokines (interferon-*γ*) and chemokines and degranulation. Lastly, the cytotoxic activity of NK cells can be increased by activation of other, less commonly mentioned receptors such as CD160 (which recognizes certain human leukocyte antigen (HLA) class I molecules) and integrins (LFA-1 or CD11a/CD18) [46]. Unfortunately, tumors have the ability to avoid NKGD2- and natural cytotoxic receptor-mediated cytotoxicity by releasing large quantities of endogenous ligands (MICA and MICB) or other factors such as transforming growth factor-*β*, indoleamine dioxygenase, and prostaglandin E2 [17, 47]. Another less common mechanism by which cancer cells can evade the attack of NK cells is through the activation of inhibitory receptors such as the killer immunoglobulin-like receptor (KIR) and NKG2A, which recognize HLA molecules [48, 49].

The function of NK cells in the perioperative period appears to be modulated by the complex interaction of several factors, including medications such as opioids, nonopioid analgesics, and volatile anesthetics; the effects of catecholamines, Th1 and Th2 cytokines, growth factors (specifically transforming growth factor-*α* and transforming growth factor-*β*), and prostaglandin E2; and the release of soluble MICA or MICB [17, 22, 50, 51]. Experimental data indicate that a common characteristic of all of these factors except for Th1 cytokines is that they reduce the killing activity of NK cells. Data from animal studies also indicate that among all of the perioperative factors involved in NK cell dysfunction, surgical stress appears to be the dominant cause. Blockade (using beta-blockers) or inhibition (using nonsteroidal anti-inflammatory drugs) of catecholamines, glucocorticoids, and prostaglandins appears to partially, if not fully, prevent the observed immune suppression [22, 52, 53].

It is important to note that the suppressive effects of glucocorticoids on NK cell cytotoxicity are observed after prolonged or repeated exposure of the NK cells to these drugs. The effects are stronger in male patients than in female patients, and the magnitude of the depression is not as substantial as that caused by catecholamines [22]. The consequence of impaired NK cell function in animals undergoing surgery is increased tumor growth, which is reduced by nonselective beta-blockers and nonsteroidal anti-inflammatory drugs; therefore, several investigators have postulated that in humans a similar phenomenon might occur [19, 53].

Clinical studies also demonstrate that the function of NK cells might be increased during surgery or immediately after. However, there is a predominant postoperative decrease that, although transient, can last up to several days or months (Figure 1) [15, 16, 54–56]. Kwon et al. demonstrated that patients who underwent surgery for hepatocellular carcinoma showed a reduction in NK cell cytotoxicity even 3 months after surgery. It has been argued that the reported reduction in NK cell function as a percentage of NK cell cytotoxicity is a result of decreases in the number of NK cells in peripheral blood mononuclear cells; however, using newer techniques of isolation and purification, we have found that NK cell cytotoxicity is still reduced [15, 55].

As previously mentioned, the function of NK cells is regulated by a tight balance between activating and inhibitory receptors. Hence, Crane et al. investigated whether the recovery of function of NK cells in patients after surgery for glioblastoma could be related to a change in the expression of the activating NKG2D receptors. The authors found that the NK cell cytotoxicity pattern of recovery was associated with an increase in NKG2D receptor expression and a decrease in circulating levels of MICA, suggesting that the decreased function observed perioperatively was a result of the release of sMICA by the tumor and consequent downregulation of the receptor [57].

Interestingly, during the perioperative period, the number of circulating tumor cells increases, mostly as a result of tumor manipulation. It has been speculated that, in favorable conditions, such as immune suppression, circulating tumor cells can seed in distant organs and form metastasis. A recent study demonstrated a possible association between NK cell function and number of circulating tumor cells. The authors speculated that impaired function in monocytes caused by low expression of toll-like receptors can be associated with poor activating signaling to NK cells, therefore leading to low NK cell cytotoxic activity [58].

Although several factors (i.e., cytokines, chemokines, growth factors, catecholamines, cortisol, and tumor-antigen release) can depress the function of the NK cells, it is possible to speculate that the complex interplay of these elements might trigger a switch in the cells from a cytolytic state toward a more immature form. Perhaps less likely, the intense activation that might occur immediately after surgical trauma might lead to exhaustion of the NK cells, as can be seen after intense exercise (Figure 2).

## 3. NK Cells and Clinical Outcomes

In patients with solid tumors, such as pancreatic or colorectal cancer, recent evidence indicates that a high number of NK cells are positively correlated with improved survival [59]. Furthermore, a favorable response to immunotherapy, as indicated by an increased number of circulating NK cells, was associated with good prognosis in patients with squamous cell carcinoma of the head and neck and in patients with ovarian cancer [60, 61]. Unfortunately, the clinical importance of an adequate NK cell count during the perioperative period is largely unknown. An observational study conducted in patients undergoing surgery for colorectal cancer showed that the preoperative NK cell count was a prognostic factor for cancer recurrence [62]. A randomized controlled trial testing the efficacy of immunotherapy in patients with colorectal cancer demonstrated that the percentage of NK cells decreased up to a month after surgery; however, although the percentage of NK cells before surgery and at the nadir was not associated with a change in survival, the percentage measured at 3 months after surgery was [32]. Importantly, patients who received immunotherapy (polysaccharide K plus tegafur or uracil) had better progression-free survival rates [32].

The function of NK cells also appears to be an important prognostic factor for survival in patients with solid tumors [62–64]. For instance, it has been suggested that patients with pancreatic or colorectal cancer who have poor NK cell cytotoxicity might have an unfavorable prognosis [63]. Tartter et al. demonstrated that preoperative NK cell cytotoxicity is a prognostic factor for cancer recurrence after surgery for colorectal cancer [62]. Another observational study showed that NK cell cytotoxicity 4 weeks after surgery for non-small-cell lung cancer was strongly associated with recurrence-free survival. Specifically, patients who showed NK cell cytotoxicity higher than 20% had the best survival compared with those who showed NK cell cytotoxicity between 10% and 20% or less than 10% [65].

In summary, the count and function of NK cells are decreased after most oncologic surgery. The duration of this immune suppression can last for several weeks and is a result of several factors, such as surgical stress and inflammatory response. Although the literature is scarce, existing evidence suggests that patients who show a significant decrease in NK cell activity or count are at increased risk for cancer recurrence and progression. Thus, it is possible to speculate that perioperative interventions targeted to restore or boost the count and function of NK cells could improve survival.

## 4. NKTT in the Perioperative Period

**Figure 3:**
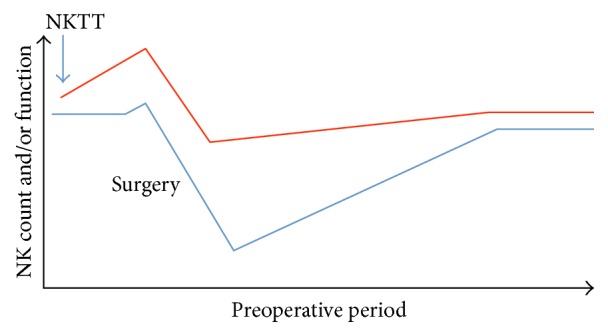
Expected changes in natural killer (NK) cell function and count after the preoperative or “preventive” infusion of expanded allogeneic or autologous NK cells (NKTT).

It is clear that immune suppression, in particular NK cell qualitative and quantitative dysfunction, is a common condition after major oncologic surgery. Therefore, the use of NKTT to overcome such immune suppression and successfully eliminate the minimal residual disease is an interesting alternative that has not yet been fully explored (Figure 3). In the following paragraphs, we will summarize the current relevant literature on NKTT in the nonsurgical setting to demonstrate why the infusion of NK cells in the perioperative period deserves further consideration.

Adoptive cell transfer therapy can be performed using patient-specific autologous, HLA-matched, allogeneic activated NK cells or commercially available NK cells such as NK-92 cells that have been expanded* in vitro* [66]. NK cells can be isolated from peripheral blood mononuclear clear cells, cord blood, bone marrow specimens, embryonic cells, or* in vitro* propagated NK cells. The NK cells are then selected and expanded with or without the addition of stimulating cytokines such as interleukin- (IL-) 2, which are artificially designed to express costimulatory molecules (IL-15 and IL-21), or genetically manipulated feeder target cells (K562 or Epstein-Barr virus-transformed lymphoblastic cells) [66–68]. One advantage of using pluripotent or embryonic stem cells as a source of NK cells over peripheral blood-derived NK cells is that stem cells can be manipulated genetically and thus improve the cytotoxic activity of the newly generated NK cells [66, 68].* Ex vivo* expanded and activated NK cells have been used against solid tumors in several clinical trials; however, these trials were phase I or II studies that were designed to test the safety and feasibility of the intervention [13, 67, 69–75].

Autologous NK cells are typically purified from patients' peripheral blood mononuclear cells, then expanded in culture medium or plasma, and stimulated under the presence of human feeder cells and IL-2. Although autologous NK cells have been administered to patients with recurrent glioma or advanced breast cancer, ovarian cancer, or melanoma, the efficacy of this intervention is still unknown owing to lack of large clinical studies assessing the actual impact on tumor progression compared with placebo [69, 72, 73]. One potential problem associated with the use of autologous NK cells for transfer therapy is that autologous NK cells can still recognize self-class I major histocompatibility complex antigens (KIR ligands) on tumor cells, which limits the cytotoxic capacity of the NK cells; therefore, the use of KIR antibodies has been suggested to overcome the inhibitory effects of KIR ligands on the newly administered NK cells and thus enhance their cytotoxicity activity [76]. Another potential problem related to the use of autologous NK cells is the risk of expanding cytokine-induced killer cells that are CD3^+^ and CD56^+^ or T-cell receptor *α*/*β* negative cells, which are less cytotoxic to NK cells [76]. Importantly, no serious adverse effects have been reported after the administration of autologous NK cells [73].

Allogeneic haploidentical NK cells have also been safely transfused into patients with solid malignancies, including metastatic melanoma and advanced renal cell cancer, non-small-cell lung cancer, breast cancer, and ovarian cancer [13, 67, 70, 71]. HLA mismatch between the donor NK cells and the tumor cells has been shown to increase the potency of the transfused NK cells [74, 75]. Good expansion and purity of NK cells have been obtained when the cells were cultured in the presence of IL-15 and hydrocortisone [71]. Both* ex vivo* and* in vivo* expansion of the donor NK cells have been attempted, with limited clinical success [70, 71]. It is worth mentioning that lymphodepletion before NK cell therapy has been recommended because it “makes space” for the donor NK cells to expand and decreases the levels of inhibitory factors [70]. Similar to autologous NK cells, allogeneic NK cells are frequently found in circulation up to 1 week after infusion; however, in some patients they have been found for longer periods of time [13, 70, 73]. Adverse reactions after the administration of allogeneic NK cells were reported as mild (grade 1) in most patients, and the adverse reactions were mostly related to the coadministration of IL-2 [71].

NK-92 cells are CD56^+^, CD3^−^, and CD16^−^ allogeneic NK cells originally isolated from non-Hodgkin lymphoma cells [66, 76, 77]. NK-92 cells lack KIR, which makes them highly cytotoxic against several cancer cell lines, including leukemia, lymphoma, melanoma, prostate cancer, and breast cancer; however, NK-92 cells are unable to mediate antibody-dependent cellular cytotoxicity [78, 79]. NK-92 cells can be continuously expanded, with doubling times of 24–36 hours, in the presence of IL-2 [76, 78]. These allogeneic cells have been safely used in humans with advanced melanoma and renal cell cancer, despite the potential concern of becoming permanently engrafted in the recipients [78]. Fever has been reported as the most common adverse reaction after infusion of NK-92 cells [79]. However, the efficacy has not been fully evaluated because most studies enrolled few patients [76–79].

Two particular populations of NK cells are the marginating-pulmonary NK cells and those residing in the liver sinusoids because of their high cytotoxic activity [80, 81]. Whether infused NK cells will remain in the pulmonary compartment or the liver is largely unknown, although it has been demonstrated that immune activating agents can efficiently modulate the function of those cells. Therefore, it is possible to speculate that the distribution and dynamics of the infused NK cells would largely depend on the predominant subset expanded* in vitro* and the interplay with adhesion molecules and chemokines on different tissues, mainly in those inflamed tissues [33, 80].

It is possible to speculate that infused NK cells could undergo suppression by the same perioperative factors that suppress endogenous NK cells. A potential solution to overcome this problem would be the infusion of* ex vivo* stimulated NK cells or the use of blockers or inhibitors of cytokines or other soluble factors that suppress the function of NK cells. For instance, the coadministration of COX inhibitors or nonselective beta-blocker during the infusion of NK cells could be seen as a feasible alternative due to the wide spread use of these medications in the perioperative period [22, 52, 53]. Anti-KIR monoclonal antibodies directed against inhibitory KIR have used in recent phase I/II trials; however, there is still little clinical experience to indicate their use in the perioperative period [82]. It is worth mentioning that stimulation of endogenous NK cells with the infusion immune-activating agent in the context of cancer surgery has not been explored in humans; however, the administration of immune activating agents such as IL-2, IL-15, or anti-PD-1 monoclonal antibodies has been associated with toxicities that can be exaggerated in the context of surgery and complicate the recovery of patients [83, 84].

In summary, there are several options for NKTT that could potentially be used in the perioperative period. Owing to the highly cytotoxic profile of NK cells and their ability to undergo expansion in large quantities, administration of NK cells derived from cord blood, induced pluripotent stem cells, or human embryonic stem cells during the perioperative period deserves further research.

## 5. Conclusion

NK cells undergo qualitative and quantitative changes in the perioperative period that point toward significant immune suppression. Although the causes of such immune dysfunction are not clear, evidence suggests that surgical stress and inflammation are the 2 main factors. The clinical consequence of a decreased number and decreased cytolytic function of NK cells appears to be of shorter postoperative survival owing to tumor progression. Therefore, strategies to preserve the quality and number of NK cells in the perioperative period are needed. NKTT is an attractive method to overcome both the deficit in function and the reduced number of NK cells because multiple sources of NK cells are available, including peripheral blood, cord blood, pluripotent stem cells, and commercially available cell lines. However, this strategy has not been tested in the perioperative period. Phase I studies to test the safety of NKTT in the context of cancer surgery are needed because cell transfer therapy could potentially improve outcomes of patients with solid tumors who undergo surgery and whose postoperative minimal residual disease could be the target of infused allogeneic or autologous NK cells.
